# Functional impact of Aurora A-mediated phosphorylation of HP1γ at serine 83 during cell cycle progression

**DOI:** 10.1186/1756-8935-6-21

**Published:** 2013-07-05

**Authors:** Adrienne Grzenda, Phoebe Leonard, Seungmae Seo, Angela J Mathison, Guillermo Urrutia, Ezequiel Calvo, Juan Iovanna, Raul Urrutia, Gwen Lomberk

**Affiliations:** 1 Laboratory of Epigenetics and Chromatin Dynamics, GIH Division, Department of Medicine, Biochemistry and Molecular Biology, Guggenheim 10, Mayo Clinic, 200 First Street SW, Rochester, MN 55905, USA; 2 Department of Obstetrics and Gynecology, Guggenheim 10, Mayo Clinic, 200 First Street SW, Rochester, MN 55905, USA; 3 Molecular Endocrinology and Oncology Research Center, Centre Hospitalier de l’Universite Laval (CHUL) Research Center, Quebec, QC G1V 4G2, Canada; 4 Institut National de la Santé et de la Recherche Médicale (INSERM), Unité 624, Stress Cellulaire, 163 Avenue de Luminy, Case 915, Parc Scientifique et Technologique de Luminy, 13288, Marseille Cedex 9, France; 5 Translational Epigenomics Program, Center for Individualized Medicine, Rochester, MN 55905, USA

**Keywords:** Heterochromatin protein 1 (HP1), Mitosis, Aurora kinase, Epigenetics, Spindle pole, Centrosome

## Abstract

**Background:**

Previous elegant studies performed in the fission yeast *Schizosaccharomyces pombe* have identified a requirement for heterochromatin protein 1 (HP1) for spindle pole formation and appropriate cell division. In mammalian cells, HP1γ has been implicated in both somatic and germ cell proliferation. High levels of HP1γ protein associate with enhanced cell proliferation and oncogenesis, while its genetic inactivation results in meiotic and mitotic failure. However, the regulation of HP1γ by kinases, critical for supporting mitotic progression, remains to be fully characterized.

**Results:**

We report for the first time that during mitotic cell division, HP1γ colocalizes and is phosphorylated at serine 83 (Ser^83^) in G_2_/M phase by Aurora A. Since Aurora A regulates both cell proliferation and mitotic aberrations, we evaluated the role of HP1γ in the regulation of these phenomena using siRNA-mediated knockdown, as well as phosphomimetic and nonphosphorylatable site-directed mutants. We found that genetic downregulation of HP1γ, which decreases the levels of phosphorylation of HP1γ at Ser^83^ (P-Ser^83^-HP1γ), results in mitotic aberrations that can be rescued by reintroducing wild type HP1γ, but not the nonphosphorylatable S83A-HP1γ mutant. In addition, proliferation assays showed that the phosphomimetic S83D-HP1γ increases 5-ethynyl-2´-deoxyuridine (EdU) incorporation, whereas the nonphosphorylatable S83A-HP1γ mutant abrogates this effect. Genome-wide expression profiling revealed that the effects of these mutants on mitotic functions are congruently reflected in G_2_/M gene expression networks in a manner that mimics the on and off states for P-Ser^83^-HP1γ.

**Conclusions:**

This is the first description of a mitotic Aurora A-HP1γ pathway, whose integrity is necessary for the execution of proper somatic cell division, providing insight into specific types of posttranslational modifications that associate to distinct functional outcomes of this important chromatin protein.

## Background

Heterochromatin protein 1 (HP1), the reader of histone H3 lysine 9 methylation (H3K9me), was originally discovered through studies in *Drosophila melanogaster* of mosaic gene silencing, known as position effect variegation [1,2]. In human and other mammalian cells, the three mammalian HP1 isoforms, HP1α, HP1β and HP1γ, have been well-studied for their localization, as well as their roles within the heterochromatic regions that associate with gene silencing. However, subsequent investigations have made it increasingly unmistakable that HP1 proteins not only localize to heterochromatic regions but also euchromatic regions [3,4]. These proteins are involved in diverse cellular processes, ranging from chromatin modification and epigenetic gene silencing to replication and DNA repair to nuclear architecture and chromosomal stability [3,4]. Moreover, HP1 proteins respond to a diversity of signaling pathways and acquire various posttranslational modifications, which impact on their function [5-9]. We have previously reported that, during interphase, phosphorylation of HP1γ at serine 83 (P-Ser^83^-HP1γ) via the cAMP-protein kinase A (PKA) pathway upon activation of cell surface receptors relocates this protein to euchromatin, where it plays a role in transcriptional elongation [8]. Thus, it is essential to define HP1-mediated pathways to map useful networks of membrane-to-chromatin signaling cascades for better understanding of the regulation of important cellular processes.

Ample evidence indicates that HP1γ is important during both somatic and germ cell proliferation. Indeed, high levels of HP1γ protein associate with enhanced somatic and meiotic cell proliferation [10]. Genetic inactivation of HP1γ results in both meiotic and mitotic failure [11,12]. Studies in primordial germ cells demonstrate that loss of HP1γ also reduces their cell number through impaired cell cycle progression [13]. However, the responsible molecular mechanisms that link this vital biological process to the functional regulation of HP1γ remain unknown.

Earlier investigations have found that HP1γ is phosphorylated throughout the cell cycle and, in particular, hyperphosphorylated in mitosis [14]. In the current study, we report a novel pathway, whereby HP1γ is regulated by mitotic kinases, in particular, Aurora kinase A, a master regulator of mitotic transitions [15]. We demonstrate that HP1γ is phosphorylated at serine 83 (Ser^83^) in G_2_/M where it colocalizes with Aurora A kinase, and its mitotic targets, cyclin B1, cyclin B2 and cyclin-dependent kinase 1 (CDK1) during cell division. HP1γ is phosphorylated at Ser^83^ by Aurora A in vitro and in cells. In addition, siRNA-mediated knockdown of HP1γ leads to a decrease of P-Ser^83^-HP1γ accompanied by mitotic aberrations. Notably, reintroduction of wild type HP1γ rescues, to a significant extent, these abnormal mitotic effects, while the nonphosphorylatable S83A-HP1γ mutant is unable to rescue this consequence of HP1γ knockdown. Congruent with these functions, phosphomimetic S83D-HP1γ results in an increase of cell proliferation, whereas the nonphosphorylatable S83A-HP1γ mutant abrogates this effect. In addition, overexpression of either the S83A-HP1γ or S83D-HP1γ mutant supports this effect in resultant cell cycle-related gene expression networks. Thus, together, these results reveal that a novel Aurora A-HP1γ pathway targeting Ser^83^ phosphorylation is necessary for the proper execution of cell division, thereby extending our knowledge of the biochemical and cell biological function of this important chromatin protein.

## Results

### HP1γ is phosphorylated at the G_2_/M phase of the cell cycle

**Figure 1:**
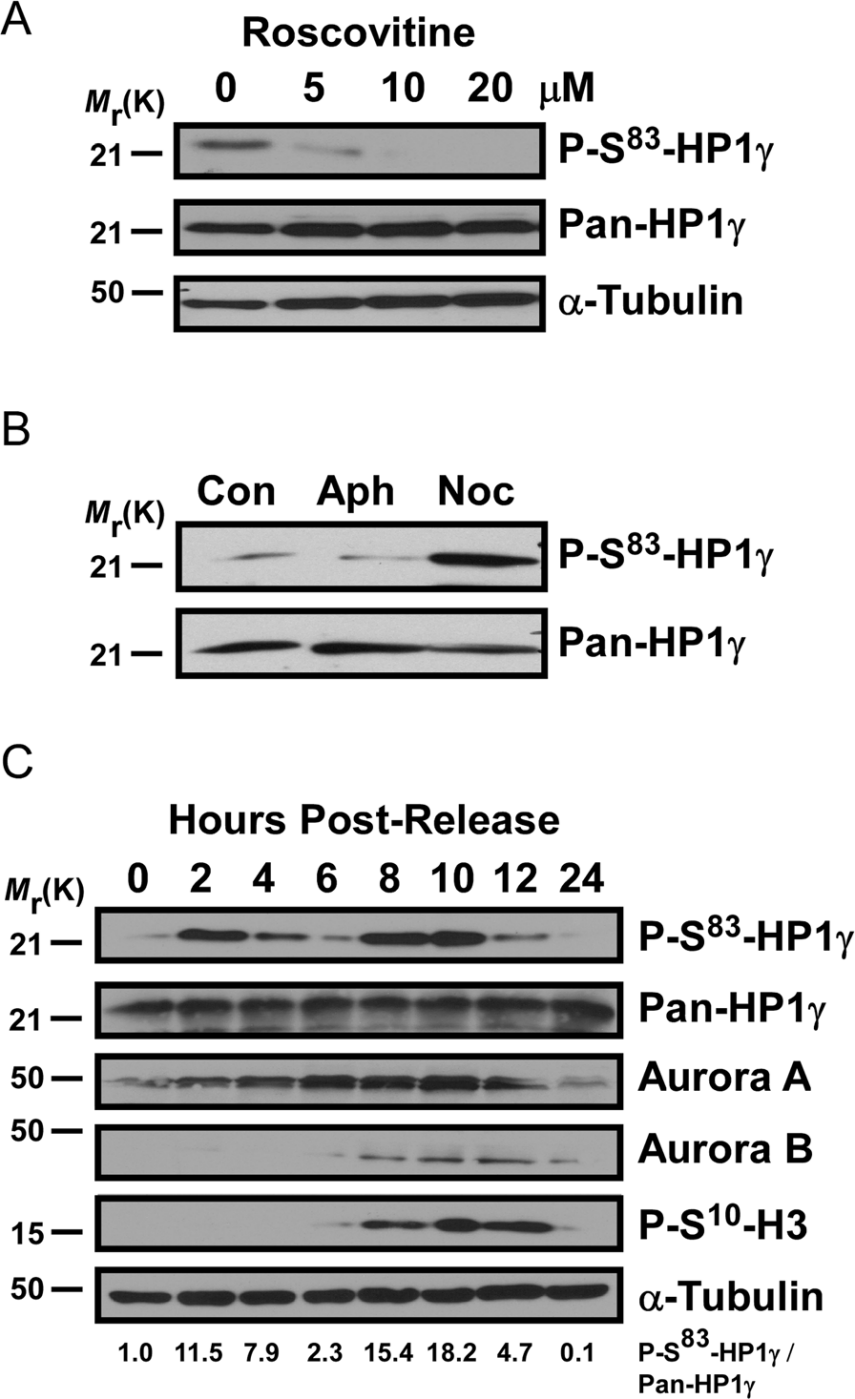
**Levels of P**-**Ser**^**83**^-**HP1γ are cell cycle**-**dependent**, **increasing significantly in G**_**2**_/**M**. **(A)** Inhibition of HP1γ phosphorylation in vivo by the cell cycle inhibitor, roscovitine. HeLa cells incubated with roscovitine, an inhibitor of cell cycle progression at the G_1_/S and G_2_/M checkpoints, display a dose-dependent inhibition of phosphorylation as shown by anti-P-Ser^83^-HP1γ (top). α-tubulin is shown as a loading control (bottom). **(B)** P-Ser^83^-HP1γ levels are high in mitotic arrested cells. Cell extracts were obtained from a normal cycling population (con), cells treated with aphidicolin (aph) to arrest cells in G_1_/S phase (G_1_/S), or mitotic-arrested cells (G_2_/M) from treatment with nocodazole (noc). An increase of P-Ser^83^-HP1γ levels in mitosis is shown by comparison of anti-P-Ser^83^-HP1γ (top) with total HP1γ (bottom). **(C)** P-Ser^83^-HP1γ levels through the cell cycle. HeLa cells were synchronized by double thymidine block and cell extracts were obtained at subsequent time points of release. P-Ser^83^-HP1γ levels are highest approximately 8 to 10 hours post-release, which corresponds to an increase in the presence of other mitotic markers, including P-Ser^10^-H3, Aurora A and Aurora B, indicating M phase entry. The relative intensity indicated below was calculated as P-Ser^83^-HP1γ/pan-HP1γ ratios and normalization with the ratio of 0 hour. aph, aphidicolin; con, control; noc, nocodazole; P-Ser^10^-H3, phosphorylation of histone H3 at serine 10; P-Ser^83^-HP1γ, phosphorylation of HP1γ at serine 83.

We have previously described that P-Ser^83^-HP1γ by PKA mediates extracellular signals during interphase [8]. In the current study, we uncover a new Aurora kinase A-mediated pathway that phosphorylates Ser^83^-HP1γ during mitosis, which is necessary for the proper execution of this process. For this purpose, we initially analyzed the kinetics of phosphorylation in HeLa cells arrested in different phases of the cell cycle. Treatment with roscovitine, a membrane permeable cyclin-dependent kinase (CDK) inhibitor, that arrests cell cycle progression at the G_1_/S and G_2_/M checkpoints [16], resulted in dose-dependent inhibition of P-Ser^83^-HP1γ (Figure 1A). To better define the temporal pattern of these events, we treated with either aphidicolin to arrest cells in S phase, or nocodazole to obtain mitotic arrest (G_2_/M). The mitotic population demonstrated a striking increase in P-Ser^83^-HP1γ levels in comparison to the normal cycling population and S phase arrested cells (Figure 1B). To define these events in the absence of kinase inhibitors, we synchronized HeLa cells by double thymidine block to obtain cell extracts at subsequent time points of release from cell cycle arrest. These experiments revealed that the levels of P-Ser^83^-HP1γ peaked twice, the first at 2 hours post-release (G_1_/S boundary, Figure 1C, Additional file 1: Figure S1 A). As this peak was likely the phosphorylation event coinciding with the previously described involvement of PKA during interphase [8], we utilized the PKA-specific inhibitor, KT5720, to treat HeLa cells upon release from double thymidine block. Upon KT5720 treatment, P-Ser^83^-HP1γ levels at 2 hours post-release were significantly diminished (Additional file 1: Figure S1 B). However, of greater interest, a more prominent second peak across 8 to 10 hours post-release from cell cycle arrest, which coincided with G_2_/M, was observed (Figure 1C, Additional file 1: Figure S1A). The lower P-Ser^83^-HP1γ levels seen in-between these two peaks (4 to 6 hours post-release, Figure 1C) corresponded with S phase (Additional file 1: Figure S1A), similar to aphidicolin treatment. These results demonstrate that levels of P-Ser^83^-HP1γ peak significantly at G_2_/M phase during the cell cycle, suggesting that phosphorylation of this protein may play a role in cell division.

**Figure 2:**
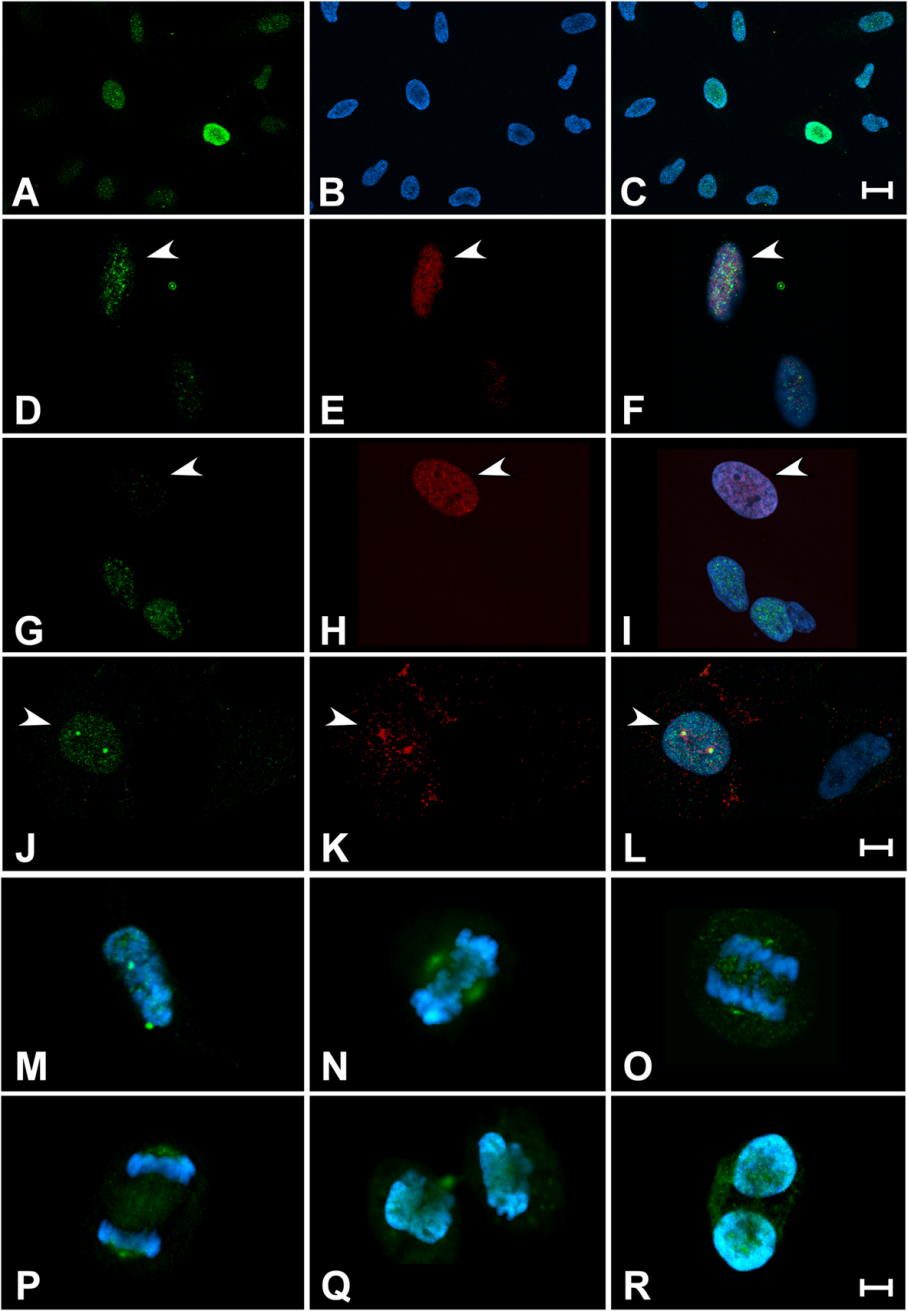
**Biphasic P-Ser**^**83**^-**HP1**γ **is observed during cell cycle progression**. **(A,B,C)** P-Ser^83^-HP1γ levels vary during the cell cycle. Panoramic view of a growing population of HeLa cells staining with anti-P-Ser^83^-HP1γ (**A**, green) demonstrates that the signal for this protein varies in intensity in different cells. Cells were counterstained with DAPI (**B**, blue) to show DNA and overlay is shown in **(C)**. Three main populations are observed according to the strength of the signal, namely strong, moderate and negligible. Scale bar represents 20 μM. **(D,E,F)** P-Ser^83^-HP1γ displays punctate euchromatic localization in G_1_ phase. Localization of P-Ser^83^-HP1γ (**D**, green) was determined in cyclin D-positive cells (**E**, red), indicative of G_1_ phase, as shown with arrows and in overlay **(F)**. **(G,H,I)** Levels of P-Ser^83^-HP1γ diminish during S phase. Negligible P-Ser^83^-HP1γ signal (**G**, green) is found in the majority of cells undergoing S phase (arrows), as determined by EdU positively labeled cells (**H**, red). Overlay is shown in **(I)**. **(J,K,L)** P-Ser^83^-HP1γ levels increase upon G_2_ entry. Cyclin B-positive cells (**K**, red), before nuclear envelope breakdown (G_2_), not only shows the P-Ser^83^-HP1γ signal (**J**, green) as a strong punctate pattern in euchromatin, but also with separating centrosomes (**L**, overlay). Scale bar represents 10 μM for panels (**D** to **L**). **(M,N,O,P,Q,R)** P-Ser^83^-HP1γ levels persist through mitosis. Cyclin B-positive, prometaphase cell demonstrates an increase in P-Ser^83^-HP1γ in association with separating centrosomes **(M)**. Metaphase cell shows the P-Ser^83^-HP1γ remains localized to centrosomes, which are forming the mitotic spindle **(N)**. Early **(O)** and late **(P)** anaphase, as well as telophase **(Q)** cells are shown, where the P-Ser^83^-HP1γ signal intensity at the centrosomes is decreased as cells prepare to complete cell division. P-Ser^83^-HP1γ signal within euchromatic regions is again observed during cytokinesis **(R)**. Scale bar represents 5 μM for panels (M to R). DAPI, 4',6-diamidino-2-phenylindole; EdU, 5-ethynyl-2´-deoxyuridine; P-Ser^83^-HP1γ, phosphorylation of HP1γ at serine 83.

Subsequently, we sought to complement the biochemical assays of phosphorylation described above by mapping the temporal pattern of staining for P-Ser^83^-HP1γ during cell cycle progression. For this purpose, we performed immunofluorescence using confocal microscopy in cells co-stained with the anti-P-Ser^83^-HP1γ and different cell cycle markers. We utilized cyclin D as a marker of G_1_, 5-ethynyl-2´-deoxyuridine (EdU)-pulse labeling for S phase, and cyclin B to indicate the G_2_ and M phases of the cell cycle. Figure 2A,B,C, which represents a low magnification field of cells stained with the anti-P-Ser^83^-HP1γ, demonstrates that the level and distribution of the signal for this modified form of HP1γ is variable in epithelial cells growing under normal conditions. Thus, we examined more carefully the levels and distribution of P-Ser^83^-HP1γ signals in relationship to key cell cycle markers. P-Ser^83^-HP1γ localization in cyclin D-positive cells (G_1_) appeared in the euchromatic compartment of the nucleus as a fine punctate pattern (Figure 2D,E,F). Quantification of cyclin D-positive cells demonstrated that 76.6% of this population (160/209) had P-Ser^83^-HP1γ staining. However, staining was relatively negligible in cells that were positively marked by a short pulse of EdU, indicative of S phase (Figure 2G,H,I) with only 22.7% of EdU-positive cells (34/150) having any P-Ser^83^-HP1γ signal. The strongest P-Ser^83^-HP1γ signal was found in 88.3% of cyclin B-positive cells (182/206), which corresponded to G_2_ (Figure 2J,K,L), and the signal continued through M in prometaphase, metaphase and anaphase, until returning to similar levels as G_1_ during telophase and cytokinesis (Figure 2M,N,O,P,Q,R). Thus, these results were congruent with our biochemical studies and confirmed that P-Ser^83^-HP1γ occurs as two peaks, beginning at G_1_ and ending at S, and the second peak which begins at G_2_ and continues during M. Interestingly, a conspicuous feature of P-Ser^83^-HP1γ localization was its staining in cyclin B-positive cells for which the nuclear membrane has not yet disassembled (late G_2_ prophase), in which the P-Ser^83^-HP1γ punctate pattern was stronger and present not only in euchromatin but also within centrosomes (Figure 2L). Although the cyclin B-positive cells found in M demonstrated reduced P-Ser^83^-HP1γ signal on chromosomes, a strong signal continued to localize at the centrosome region of the mitotic spindle (Figure 2M,N,O,P). In all these cases, P-Ser^83^-HP1γ coincided with the presence of cyclin B at the centrosome. As several mitotic kinases are highly enriched at this organelle [17], these studies prompted us to identify the kinase responsible for the significant P-Ser^83^-HP1γ event found during mitotic progression.

### HP1γ is phosphorylated at G_2_/M by Aurora A

**Figure 3:**
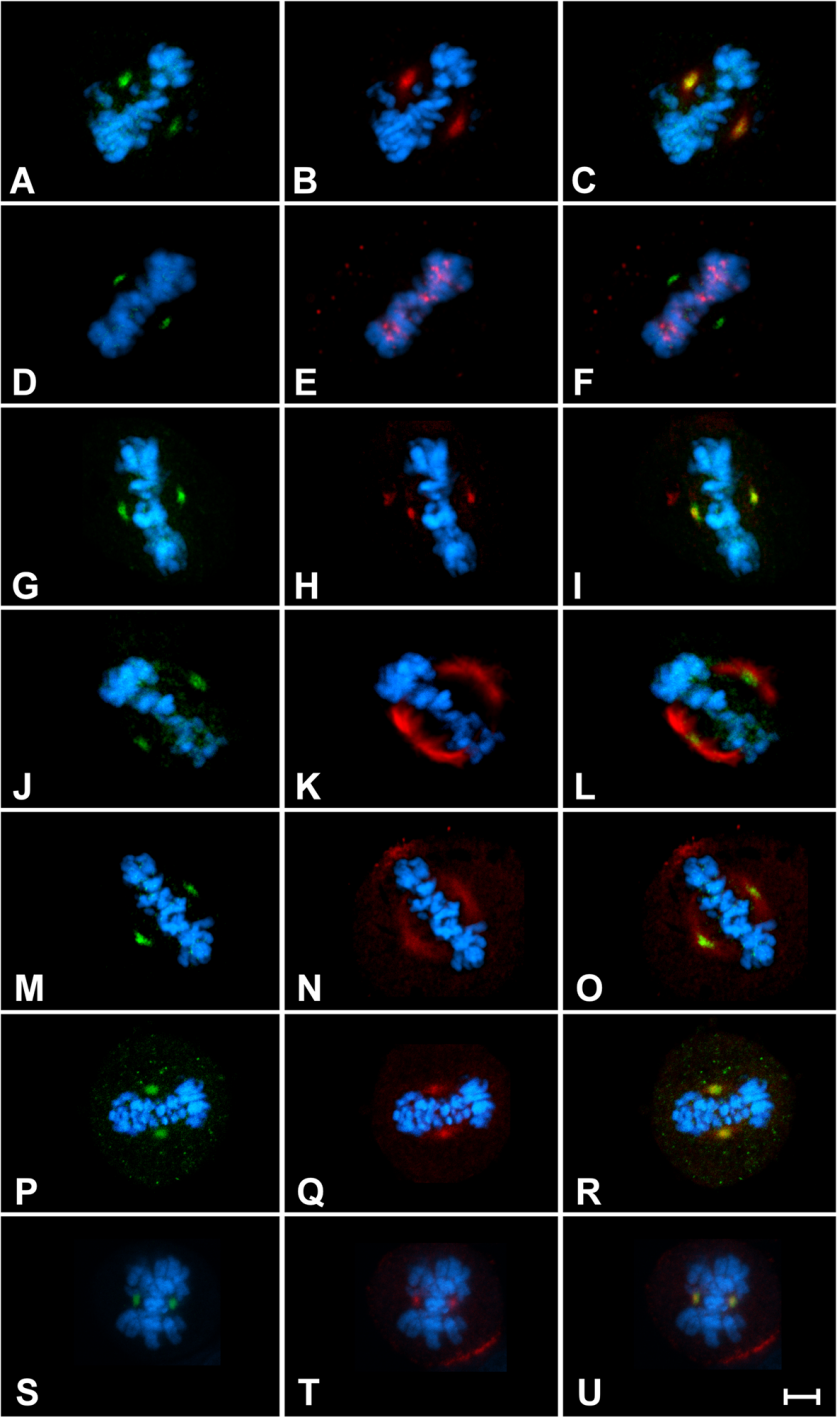
**P**-**Ser**^**83**^-**HP1γ colocalizes with Aurora A at the mitotic spindle**. Representative images are shown for localization in mitotic HeLa cells. **(A,B,C)** Colocalization of P-Ser^83^-HP1γ (**A**, green) is shown with Aurora A (**B**, red) at the spindle poles. The overlay is shown in **(C)**. **(D,E,F)** Cells in metaphase were also stained for P-Ser^83^-HP1γ (**D**, green) and Aurora B (**E**, red), which demonstrates that there is no colocalization of these two proteins as observed in the overlay **(F)**. **(G,H,I,J,K,L)** P-Ser^83^-HP1γ (**G**,**J**, green) was confirmed to be present at the spindle poles through co-staining with γ-tubulin (**H**, red) as well as α-tubulin (**K**, red) as shown in the overlays (**I**, **L**). **(M,N,O,P,Q,R,S,T,U)** In addition, CDK1 (**N**, red), cyclin B1 (**Q**, red) and cyclin B2 (**T**, red) were each shown to co-localize with P-Ser^83^-HP1γ (**M**,**P**,**S**, green) as shown by overlays (**O**,**R**,**U**). Cells were counterstained with DAPI (blue) to show DNA. Scale bar represents 5 μM. CDK1, cyclin-dependent kinase 1; DAPI, 4',6-diamidino-2-phenylindole; P-Ser^83^-HP1γ, phosphorylation of HP1γ at serine 83.

While PKA was implicated in the first peak of P-Ser^83^-HP1γ levels that occur at G_1_, the kinase that mediates the second peak of P-Ser^83^-HP1γ at G_2_/M, described here, remained unknown. Interestingly, we found that the temporal pattern of P-Ser^83^-HP1γ coincided with phosphorylation of histone H3 at serine 10 (P-Ser^10^-H3, Figure 1C). P-Ser^10^-H3 initiates during G_2_ in pericentric foci and spreads along the chromosome arms, thus serving as a hallmark of mitosis [18]. Previously derived consensus sequences for Aurora kinases suggested that, similar to P-Ser^10^-H3, Ser^83^-HP1γ might be a target of Aurora kinases [19]. Additional experiments demonstrated that the temporal pattern of P-Ser^83^-HP1γ was similar to both Aurora A and Aurora B (Figure 1C). These results led us to hypothesize that the newly described P-Ser^83^-HP1γ at G_2_/M was achieved through the activity of an Aurora kinase. Thus, we first performed immunofluorescence experiments to determine whether P-Ser^83^-HP1γ co-localized with any of these kinases at G_2_/M. Indeed, we found that P-Ser^83^-HP1γ localized to areas rich in Aurora A (Figure 3A,B,C), but not Aurora B (Figure 3D,E,F). P-Ser^83^-HP1γ was also confirmed to be present at the Aurora A-rich area of the spindle poles through colocalization with γ-tubulin (Figure 3G,H,I) and α-tubulin (Figure 3J,K,L). More importantly, we found that critical regulators of G_2_/M progression, which are also targets of Aurora A, namely cyclin B1, cyclin B2 and their partner kinase, CDK1, also colocalized with P-Ser^83^-HP1γ (Figure 3M,N,O,P,Q,U). Together, these results demonstrated that mitotic phosphorylation confers a distinct localization of this HP1γ subpopulation to the spindle poles that is marked by the G_2_/M Aurora A-cyclin B-CDK1 pathway, supporting the idea that this kinase may be the enzyme involved in P-Ser^83^-HP1γ at G_2_/M.

**Figure 4:**
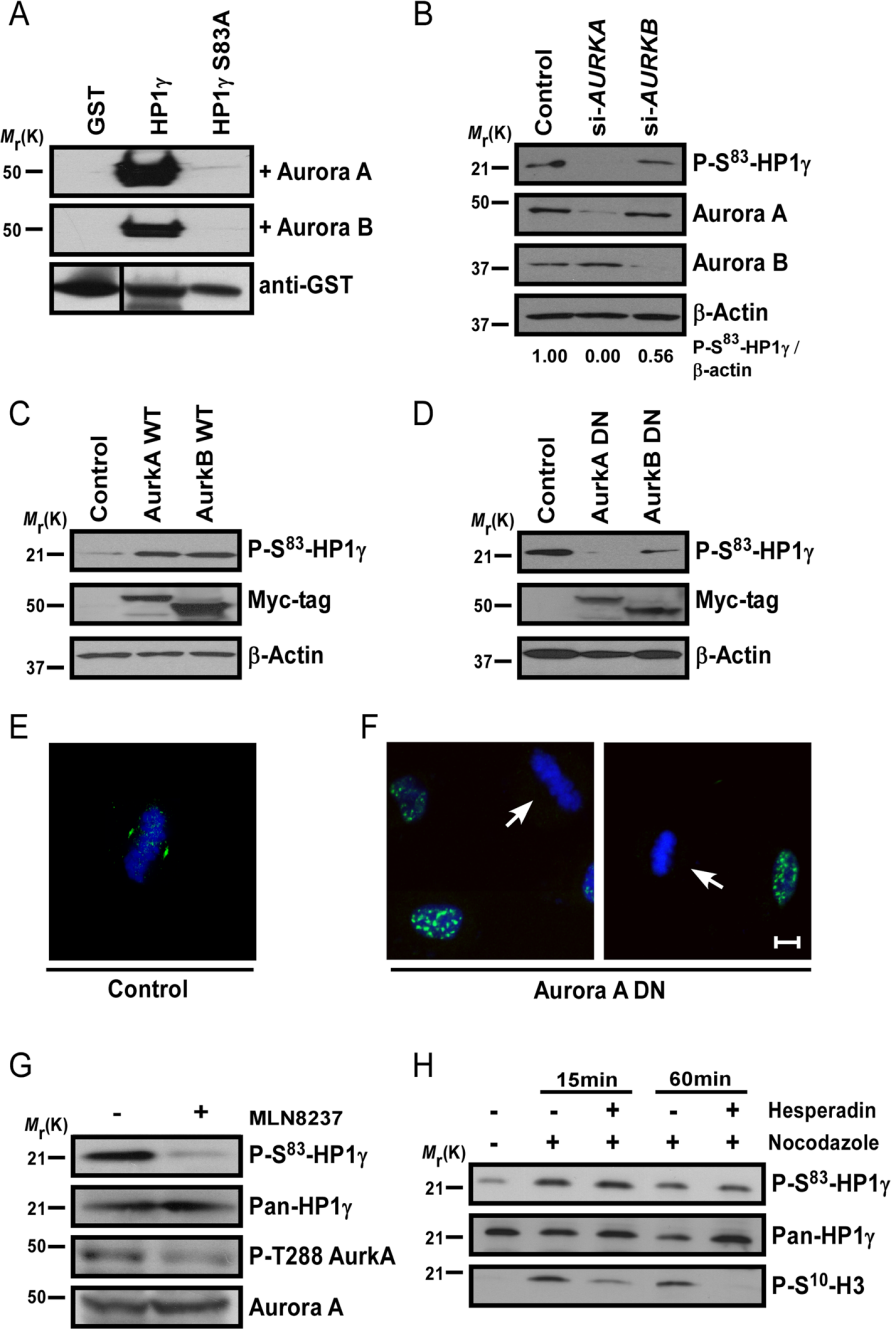
**Aurora A phosphorylates Ser**^**83**^-**HP1γ in G**_**2**_/**M**. **(A)** Aurora kinases phosphorylate Ser^83^ in vitro. In vitro kinase assays were performed on GST fusion proteins, which demonstrate that wild type, not S83A-HP1γ mutant, is phosphorylated by Aurora kinases. **(B)** Aurora A siRNA reduces P-Ser^83^-HP1γ. Aurora A siRNA significantly reduced P-Ser^83^-HP1γ, whereas Aurora B siRNA only slightly reduced P-Ser^83^-HP1γ (top). Aurora A (*AURKA*) and Aurora B (*AURKB*) were effectively knocked-down (middle panels). Relative intensities were calculated as P-Ser^83^-HP1γ/β-actin ratios. **(C)** Wild type Aurora kinases increase P-Ser^83^-HP1γ. CHO cells, with low basal P-Ser^83^-HP1γ, demonstrated increased P-Ser^83^-HP1γ (top) upon transfection of Aurora kinases (Myc-tag; middle). **(D)** Aurora A-dominant negative (DN) reduces P-Ser^83^-HP1γ. P-Ser^83^-HP1γ (top) was significantly reduced with Aurora A-DN in BxPC3, epithelial cells with high basal P-Ser^83^-HP1γ. Aurora B-DN also reduced P-Ser^83^-HP1γ, although still detected. Aurora-DN levels are shown by Myc-tag. β-actin serves as loading control (B, C, D; bottom). **(E,F)** Aurora A-DN abolishes mitotic P-Ser^83^-HP1γ. Representative images of overlays with DAPI counterstain are shown for P-Ser^83^-HP1γ (green) with control (E) or Aurora A-DN (F). Typical P-Ser^83^-HP1γ localization was still observed in interphase with Aurora A-DN, but disrupted in metaphase (arrows). Scale bar represents 10 μM. **(G,H)**. Pharmacological inhibition of Aurora A, but not Aurora B, inhibits P-Ser^83^-HP1γ. Aurora A inhibition with MLN8237 was confirmed by loss of activated P-Thr^288^ relative to total Aurora A (G, lower panels). P-Ser^83^-HP1γ was significantly reduced with MLN8237, without affecting pan-HP1γ (G, upper panels). Conversely, Aurora B inhibition by hesperidin did not reduce P-Ser^83^-HP1γ (H, top). Aurora B inhibition was confirmed by P-Ser^10^-H3, a well-known Aurora B target (H, bottom). CHO, Chinese hamster ovary; DAPI, 4',6-diamidino-2-phenylindole; DN, dominant negative; GST, glutathione S-transferase; P-Ser^10^-H3, phosphorylation of histone H3 at serine 10; P-Ser^83^-HP1γ, phosphorylation of HP1γ at serine 83; P-Thr^288^, phosphorylation of Aurora A at threonine 288; Ser^83^, serine 83.

To mechanistically test this hypothesis, we initially incubated glutathione S-transferase (GST) fusion wild type and nonphosphorylatable mutant HP1γ proteins with each Aurora kinase, Aurora A or Aurora B, followed by western blot using the phospho-specific P-Ser^83^-HP1γ antibody. These in vitro kinase assays demonstrated that the wild type HP1γ, but not the dominant negative, nonphosphorylatable S83A-HP1γ mutant [8], could be phosphorylated in vitro by both Aurora A and Aurora B (Figure 4A). To determine whether Aurora kinases also phosphorylate HP1γ in vivo, we performed western blots of siRNA-treated HeLa cells against Aurora A and B, separately (Figure 4B). We found that Aurora A siRNA can inhibit the P-Ser^83^-HP1γ in vivo, whereas Aurora B siRNA demonstrated only a slight reduction in levels of P-Ser^83^-HP1γ (56% of control levels). Of note, Aurora A kinase depletion by siRNA also leads to arrest of cells at G_2_/M [20], thus eliminating the influence of the G_1_ phosphorylation in these experiments. To further investigate the participation of Auroras in this event, Chinese hamster ovary (CHO) cells, which have relatively low basal levels of P-Ser^83^-HP1γ, were transfected with either wild type Aurora A or Aurora B (Figure 4C). As a result, levels of P-Ser^83^-HP1γ were higher in the Aurora-transfected cells than control. This occurred with both Aurora A and Aurora B transfection, as expected due to their effects on cell cycle progression. In contrast, transfection of epithelial cells, BxPC3, which have high basal levels of P-Ser^83^-HP1γ, with the dominant negative form of Aurora A [21,22] resulted in reduced levels of P-Ser^83^-HP1γ (Figure 4D). Similar to the siRNA experiments, dominant negative Aurora B [21,23] had less effect on P-Ser^83^-HP1γ levels than Aurora A. Therefore, we utilized the dominant negative Aurora A in HeLa cells to confirm this phenomenon by immunofluorescence. Compared to control cells (Figure 4E), transfections with dominant negative Aurora A (Figure 4F) abolished the localization of the P-Ser^83^-HP1γ in cell compartments rich in this kinase (arrow). In contrast, transfection with dominant negative Aurora A did not affect P-Ser^83^-HP1γ levels or localization in interphase cells (Figure 4F). Furthermore, we utilized the Aurora A- and Aurora B-specific pharmacological inhibitors, MLN8237 and hesperidin, respectively, to determine the participation of each kinase in P-Ser^83^-HP1γ. We found that specific inhibition of Aurora A with 300 nM MLN8237, which was confirmed by loss of activated phosphorylation of Aurora A at threonine 288 (P-Thr^288^) [24], diminished P-Ser^83^-HP1γ levels without affecting total HP1γ protein levels (Figure 4G). However, treatment with hesperidin (200 nM) for specific inhibition of Aurora B did not reduce P-Ser^83^-HP1γ levels, while still inhibiting the Aurora B target P-Ser^10^-H3 (Figure 4H, and personal communication with H Dormann and CD Allis). Combined, these results demonstrate that Aurora A kinase is primarily responsible for the localization and increased level of P-Ser^83^-HP1γ observed in G_2_/M. Together with the biochemical experiments described above, these data implicate, for the first time, Aurora kinase in the cell cycle-regulated P-Ser^83^-HP1γ. This observation also represents the first evidence describing mammalian HP1 at the spindle poles, a localization that is preferred by a large amount of proteins involved in the regulation of cell cycle transitions.

### P-Ser^83^-HP1γ is required for normal mitotic function

**Figure 5:**
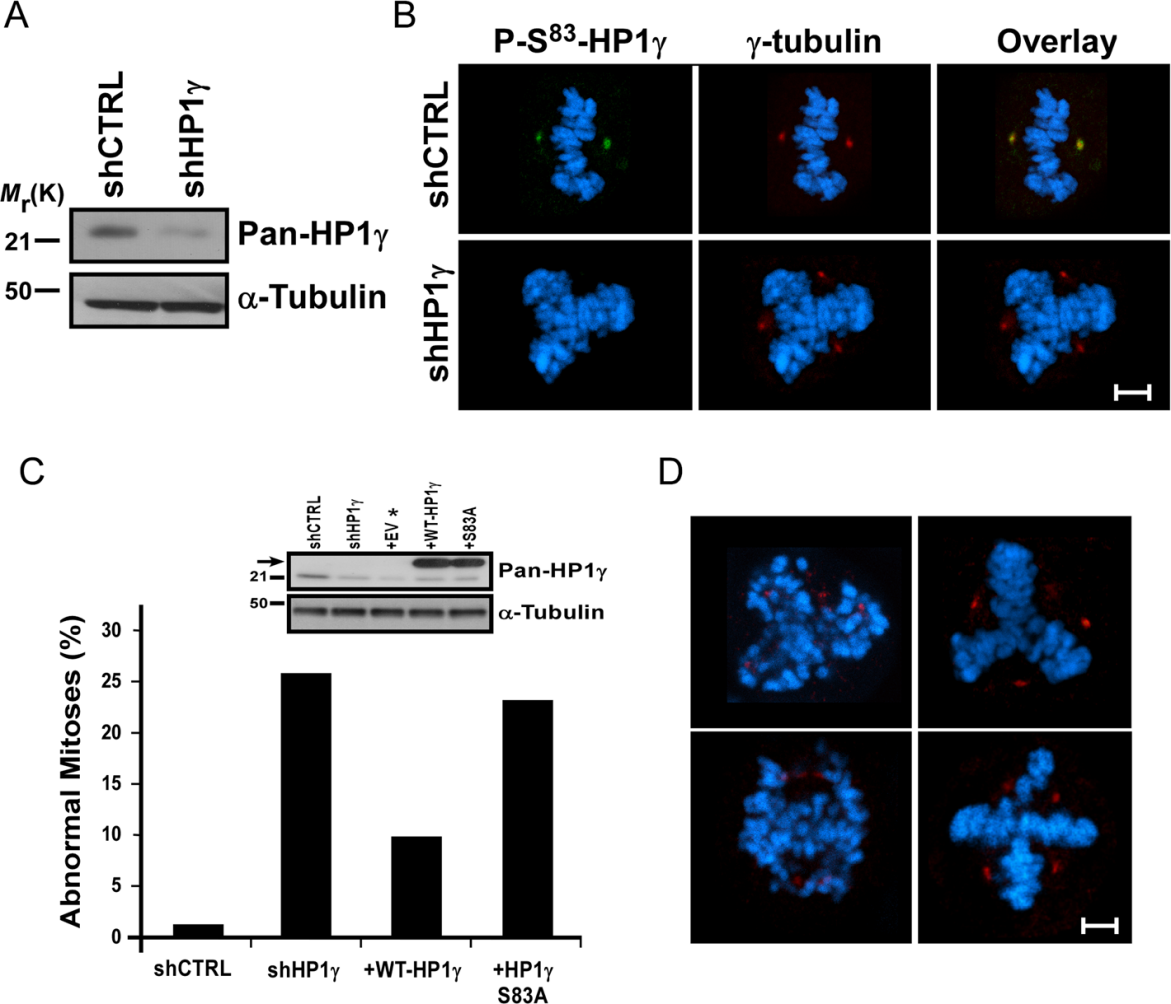
**P-Ser**^**83**^-**HP1γ is necessary for proper mitotic function**. **(A)** Stable knockdown of HP1γ in HeLa cells. Western blot of HP1γ levels (top) is shown from HeLa cell lysates to confirm stable lentiviral-mediated shHP1γ compared to shCTRL. α-tubulin serves as a loading control (bottom). **(B)** HP1γ knockdown eliminates P-Ser^83^-HP1γ at the spindle poles. Representative images are shown for immunofluorescence on shCTRL and shHP1γ HeLa cells to demonstrate specific loss of P-Ser^83^-HP1γ (green) staining. Co-staining with γ-tubulin (red) was performed to establish the localization of the spindle poles. Cells were counterstained with DAPI and the overlay is shown. Scale bar represents 5 μM. (**C**) Mitotic aberrations caused by HP1γ knockdown are rescued by wild type, but not S83A-HP1γ mutant. Mitotic aberrations were quantified for shCTRL and shHP1γ cells. In order to determine if Ser^83^ phosphorylation plays a role in this function, shHP1γ cells were infected with adenovirus carrying wild type or S83A-HP1γ mutant. While reintroduction of wild type HP1γ was able to significantly rescue this effect, S83A-HP1γ mutant was not, implicating Aurora A-mediated phosphorylation in this phenomenon. For each condition, 200 mitotic cells were analyzed. Western blot is shown of endogenous HP1γ levels (inlay, top) as well as transduced His-tagged wild type and S83A-HP1γ mutant proteins (arrow). α-tubulin serves as a loading control (inlay, bottom). *Transduction with EV control did not change the number of abnormalities observed with shHP1γ. **(D)** Mitotic aberrations observed in stable shHP1γ cells include multipolar spindles, centrosome disruption and lagging, unorganized chromosomes. Representative images are shown for the types of observed mitotic aberrations. γ-tubulin (red) marks spindle poles with DAPI counterstain to show condensed mitotic chromosomes. Scale bar represents 5 μM. DAPI, 4',6-diamidino-2-phenylindole; EV, empty vector; P-Ser^83^-HP1γ, phosphorylation of HP1γ at serine 83; Ser^83^, serine 83; shCTRL, shRNA control; shHP1γ, shRNA knockdown of HP1γ; shRNA, short hairpin RNA.

Functionally, HP1γ has been previously shown to play a role in cell cycle progression [10-13], although how this protein is regulated to modulate this function remains unknown. Inhibition of Aurora A leads to mitotic spindle defects and misaligned chromosomes [25,26]. Thus, as phosphorylation of HP1γ is downstream of this pathway during mitosis, we investigated whether disrupting the function of this protein also coincides with this effect. For this purpose, we performed stable lentiviral-mediated shRNA knockdown of HP1γ (shHP1γ) in HeLa cells. HP1γ knockdown was confirmed by western blot with approximately 90% reduction in protein levels (Figure 5A). These cells also displayed a significant decrease in P-Ser^83^-HP1γ staining by immunofluorescence (Figure 5B), demonstrating that localization of P-Ser^83^-HP1γ to the mitotic spindle pole was unambiguous. We found that 25.5% of shHP1γ cells in mitosis displayed abnormalities (n = 200, Figure 5C), including multipolar spindles, centrosome disruption or lagging, unorganized chromosomes (Figure 5D). shRNA control cells (shCTRL) displayed abnormalities in only 1% (n = 200). However, in spite of this informative outcome, since HP1γ knockdown depleted all forms of the protein, the contribution of Ser^83^ phosphorylation to this effect could not be assessed by this experimental manipulation. Thus, to better determine the role that phosphorylation of Ser^83^ plays in this function, we sought to rescue the knockdown phenotype with wild type and Ser^83^ mutant HP1γ. Transduction with empty vector (EV) control did not change the number of abnormalities observed with shHP1γ. Reintroduction of wild type HP1γ (+WT-HP1γ) rescued, to a significant extent, the abnormal mitotic effects seen with knockdown of this protein (10% abnormal, n = 200). Notably, an alanine substitution, which rendered HP1γ unable to undergo phosphorylation at Ser^83^ (+S83A), was unable to rescue the knockdown phenotype (23% abnormal, n = 200). This data indicates that first, normal HP1γ levels are necessary for proper mitotic functions and second, HP1γ must be amenable to Aurora A-mediated Ser^83^ phosphorylation to achieve these effects.

### P-Ser^83^-HP1γ status affects cell proliferation and mitotic gene expression networks

**Figure 6:**
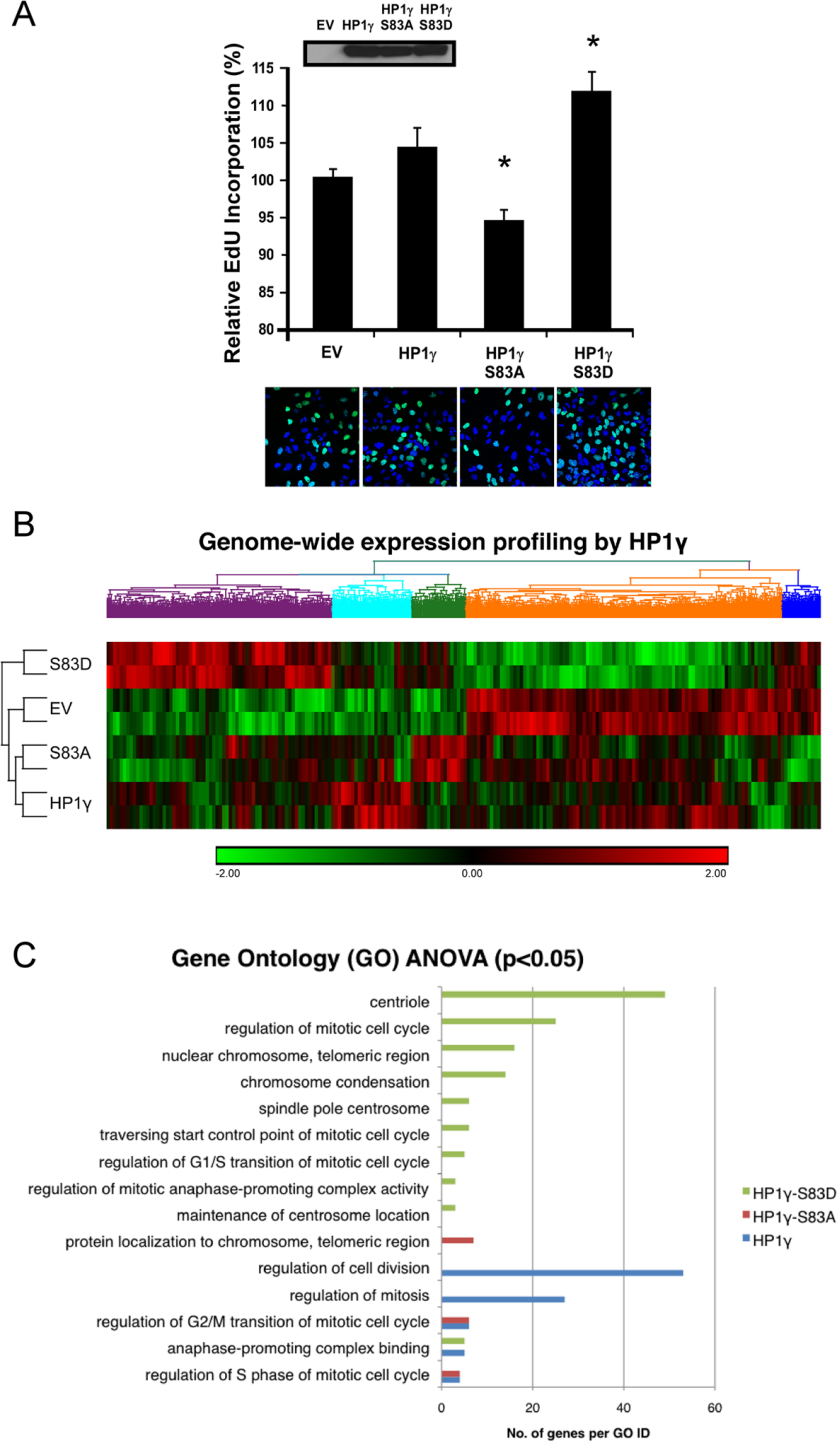
**P-Ser**^**83**^-**HP1γ status alters cell proliferation and cell cycle**-**related gene networks**. **(A)** P-Ser^83^-HP1γ plays a role in cell proliferation. Cell proliferation was measured in the presence of control (EV), wild type HP1γ, the nonphosphorylatable (S83A)- or phosphomimetic (S83D)-HP1γ mutants by EdU incorporation, using both FACS and microscopy. Wild type HP1γ demonstrated only a slight increase in EdU incorporation compared to EV. However, while mutation of S83A-HP1γ decreased the levels of EdU, the S83D-HP1γ mutant had a significant increase in levels of EdU incorporation over control cells. Western blot controlling expression of His-tagged wild type and mutant HP1γ proteins is shown (top, inlay). A representative immunofluorescence image (40 × magnification) of EdU-positive cells (green) is shown below each respective experimental condition. Cells were counterstained with DAPI to detect total number of cells present in a field. *P values <0.05. **(B)** Genome-wide expression analysis of HP1γ highlights consequences of Ser^83^ phosphorylation. Hierarchical clustering of significant targets (*P* value <0.05) from Affymetrix Human Gene 1.0 ST microarray demonstrates the close relationship between EV and the nonphosphorylatable S83A-HP1γ mutant. Large clusters of genes show deregulation in the presence of either the nonphosphorylatable (S83A)- or phosphomimetic (S83D)-HP1γ mutants. **(C)** P-Ser^83^-HP1γ status influences the expression of G_2_/M-related genes. Gene Ontology (GO) ANOVA reveals significant differential expression of genes by both wild type and mutant HP1γ in functional groupings related to mitosis and cell division, again indicating that the presence of an active phosphorylation site at Ser^83^ is necessary for proper mitotic function as a sizeable number of targets are deregulated in the presence of the HP1γ mutants with altered phosphorylation abilities. ANOVA, analysis of variance; DAPI, 4',6-diamidino-2-phenylindole; EdU, 5-ethynyl-2´-deoxyuridine; EV, empty vector; FACS, fluorescence-activated cell sorting; GO, Gene Ontology; P-Ser^83^-HP1γ, phosphorylation of HP1γ at serine 83; Ser^83^, serine 83.

Normal mitotic cell division is a prerequisite for proliferative homeostasis and proper cell cycle progression [27]. Thus, based on our results demonstrating the role of P-Ser^83^-HP1γ in mitosis, we examined the resultant effects of P-Ser^83^-HP1γ on cell proliferation by analyzing cells transfected with wild type, S83A-HP1γ or S83D-HP1γ mutant via EdU incorporation. We found that wild type HP1γ had a slight increase in EdU incorporation compared to EV control (103.9% ± 2.6% of EV control, Figure 6A). However, nonphosphorylatable S83A-HP1γ mutant decreased the levels of EdU (94.2% ± 1.6% of EV control, *P* <0.05, Figure 6A). Notably, an aspartic acid substitution (S83D), designed to mimic Ser^83^ phosphorylation, had a significant increase in levels of EdU incorporation over control cells (111.2% ± 2.6% of EV control, *P* <0.05, Figure 6A). Thus, these results support the idea that phosphorylation of Ser^83^ is necessary for the regulation of cell cycle progression by HP1γ.

We next investigated whether the changes observed in EdU incorporation by both phosphomimetic and nonphosphorylatable Ser^83^-HP1γ mutants were accompanied by changes in other biochemical surrogates for cell cycle progression, such as known mitotic gene networks. For this purpose, we performed a genome-wide query using Affymetrix (Santa Clara, CA, USA) profiles as transcriptional readouts of their effects. Hierarchical clustering of targets significantly altered by HP1γ (526 targets), S83A-HP1γ (492 targets) or S83D-HP1γ (1,727 targets) overexpression demonstrated that gene networks modulated by HP1γ experienced deregulation in the presence of the Ser^83^ mutation, indicating dependence of these processes on regulation of Ser^83^ phosphorylation (Figure 6B). Based on Euclidean distance calculation and the resulting dendrogram, both control and nonphosphorylatable S83A-HP1γ mutant samples were statistically the most similar (Figure 6B). The fact that the EV and the S83A-HP1γ mutant possessed the closest relationship suggested that the latter worked predominantly as either an inactive or dominant negative mutant. However, the phosphomimetic S83D-HP1γ mutant, for the most part, reversed the effect of the S83A-HP1γ mutant, suggesting that it likely worked in a constitutively active manner thereby mimicking Aurora A-mediated Ser^83^ phosphorylation. Pathway-specific RT-PCR was used to validate a subset of significant targets (Additional file 2: Table S1). These experiments revealed that HP1γ and its phosphorylated form have the ability to change the levels of transcripts related to mitosis.

Gene Ontology (GO) ANOVA analysis was utilized to probe for differentially expressed functional groupings of genes (Figure 6C). Overall, HP1γ overexpression resulted in significant enrichment of targets related to regulation of cellular proliferation, cell division, and mitosis (*P* <0.05). S83A-HP1γ mutant overexpression yielded differential expression in targets related to protein localization to the chromosome, regulation of the S phase of the mitotic cell cycle and regulation of the G_2_/M transition of mitotic cell cycle. S83D-HP1γ mutant overexpression showed significant alteration in genes related to the regulation of the mitotic cell cycle, regulation of the G_2_/M anaphase-promoting complex, maintenance of centrosome location and spindle pole structure, among others. Consequently, from these data, we conclude that disruption of phosphorylation status of HP1γ has diverse effects on multiple aspects of the mitotic cell cycle, which is congruent with its cell cycle-associated phosphorylation pattern (Figures 1 and 2) indicating a pervasive role of the regulation of HP1γ in cell division.

Interestingly, previous studies have shown that depletion of HP1γ in primordial germ cells reduces their number as a result of impaired cell cycle progression [13]. Comparison of our expression dataset with a published dataset in primordial germ cells revealed that the expression of the nonphosphorylatable S83A-HP1γ mutant displayed a highly similar pattern as HP1γ depletion, including targets related to cell cycle, proliferation and growth. This ability of the S83A-HP1γ mutation to mimic conditions of absolute HP1γ depletion at the level of gene expression networks, combined with the inability of the S83A-HP1γ mutant to rescue the mitotic defects observed with HP1γ knockdown, indicates that posttranslational modification of this residue is needed for proper progression through mitosis. Furthermore, it may be concluded from our genome-wide analysis that HP1γ participates in the regulation of processes, which support proper cell division and proliferation through phosphorylation-dependent and phosphorylation-independent mechanisms.

## Discussion

Based on the current study, our demonstration that HP1γ, a well-known epigenetic regulator, undergoes robust phosphorylation at Ser^83^ in G_2_/M has significant biological relevance and deserves careful consideration. Previous studies demonstrating that HP1 proteins are ejected from chromosomes during mitosis [28,29] led to the assumption that this protein is not involved in the regulation of this process, even though it is highly express in rapidly dividing cancer cells [10]. In this regard, the current study reveals that, during G_2_/M, an extrachromosomal subpopulation of HP1γ, P-Ser^83^-HP1γ, localizes with γ-tubulin, Aurora A kinase and other mitotic targets, including cyclin B1, cyclin B2 and CDK1, at the spindle poles. Thus, this data demonstrates for the first time that, in spite of its ejection from chromosomes, HP1γ does not disappear during mitosis, but rather relocates to organelles, known for enrichment in cell cycle regulators, where it undergoes G_2_/M-specific phosphorylation at Ser^83^ by Aurora A. In addition, the colocalization and coupling of Aurora A to HP1γ in cell cycle regulation is reconstituted in time and space in each cell cycle.

Examination of the effect of the related kinase, Aurora B, demonstrates that this enzyme can phosphorylate the Ser^83^ site in vitro. However, siRNA and dominant negative experiments demonstrate that Aurora B was not as robust as Aurora A on modulating levels of P-Ser^83^-HP1γ in cells. Treatment of cells with the Aurora B inhibitor, hesperidin, does not impair P-Ser^83^-HP1γ and, more importantly, Aurora B does not localize with P-Ser^83^-HP1γ in mitotic cells. These results reveal a significant level of specificity for these kinases in the phosphorylation of HP1 proteins.

We found that HP1γ, though ejected from chromosomes by the previously described Aurora-mediated P-Ser^10^-H3 [28,29], remains tightly associated to a mitotic organelle which is rich in cell cycle regulators. This reveals the existence of coupled mechanisms of ejection and relocalization of HP1γ, which ultimately has significant consequences for the regulation of cell division. Both steps involved in this process, H3 and HP1γ phosphorylation, are mediated by Aurora kinases. Thus, it is most likely that one function of Auroras has evolved, in part, to secure that epigenetic regulators are turned on and off during cell division in a highly synchronized manner, to achieve the proper transfer of genetic-epigenetic material through generations. Interestingly, although HP1 proteins themselves have not been previously observed at the centrosome/spindle pole, several HP1-interacting proteins are known to reside in this cell compartment. For example, a subpopulation of origin recognition complex 2 (Orc2) protein has been localized to centrosomes [30]. However, contrary to the Aurora A-cyclin B-CDK1 pathway, which links the phosphorylation of HP1γ at the spindle during G_2_/M transition, Orc2 associates with HP1 only in the population that is tightly bound to heterochromatin in G_1_ and early S phase. In addition, immunoprecipitation of Orc2 shows specific interaction with HP1α and HP1β, but not HP1γ [30], the HP1 protein studied here. Since posttranslational modifications of HP1 were not considered in the Orc2 experiments, it remains possible that subpopulations of distinct posttranslationally modified HP1 proteins, such as P-Ser^83^-HP1γ, which cannot be detected with pan-HP1 antibodies, also interact with Orc2. It is not likely, however, that Orc2 is responsible for recruitment of HP1γ to this cell compartment, given that Orc2 is localized there throughout the entire cell cycle [30]. Nevertheless, our results demonstrate a high degree of selectivity for HP1γ to work with certain regulatory enzymes (kinases) to maintain mitotic functions.

Previous studies have shown that disruption of G9a, one of the histone methyltransferases responsible for the histone mark recognized and bound by HP1, H3 lysine 9, results in chromosome instability along with centrosome abnormalities [31]. In addition to creating the mark to which HP1 binds, G9a localizes with HP1α and HP1γ, which is dependent upon its own automethylation [32], and HP1γ has been shown to specifically form complexes with G9a in the context of the E2F-6 gene silencing complex [33]. Interestingly, in meiosis cell division during gamete production, HP1γ and G9a are proposed to form an axis that is responsible for retaining centromeric regions of unpaired homologous chromosomes in close alignment, and facilitating progression of their pairing in early meiotic prophase [12]. In fact, HP1γ-deficient mouse spermatocytes undergo meiotic catastrophe [12]. An important observation of our studies is that siRNA-mediated knockdown of HP1γ leads to a decrease of P-Ser^83^-HP1γ accompanied by mitotic aberrations. While reintroduction of wild type HP1γ rescues, to a significant extent, these abnormal mitotic effects, the nonphosphorylatable S83A-HP1γ mutant is unable to rescue this consequence of HP1γ knockdown, highlighting the importance of Ser^83^ modification for this function. Moreover, the S83D-HP1γ mutant that mimics Aurora A phosphorylation facilitates cell proliferation, whereas the nonphosphorylatable S83A-HP1γ mutant inhibits this process. Therefore, it is tempting to speculate whether modifications of HP1 influence interactions with G9a and whether these proteins function together in regulating proper cell division. Indeed, additional studies using model organisms support that the function described here for human HP1 proteins is conserved. In *Schizosaccharomyces pombe*, the HP1 homologue, Swi6, is required to preserve genomic integrity and proper segregation of chromosomes during mitosis [34]. Impaired Swi6 function leads to mitotic alterations that cause severe growth alterations. Furthermore, the HP1-like protein in *Dictyostelium discoideum*, AX4 chromo domain-containing protein (hcpA), which displays 79% similarity to human HP1γ, colocalizes with electron-dense structures at the nuclear periphery that are compatible with pericentrosomal material [35]. Overexpression of this protein causes growth defects that are accompanied by an increase in the frequency of atypical anaphase bridges. Genetic studies in *Drosophila* have demonstrated that mutations in the HP1 protein cause defective chromosome segregation [36,37]. Thus, in combination with this data, the studies described here indicate that HP1 proteins have evolved to support cell division in organisms ranging from fission yeast to humans.

Congruent with our results, previous experiments have defined a role for HP1γ in human diseases that are characterized by abnormal cell proliferation. High levels of HP1γ have been observed in several cancer types, including esophageal, breast, colon, lung and cervical cancer, the cell model used here [10]. In addition, siRNA-mediated knockdown of HP1γ expression inhibits cervical cancer cell proliferation. Of note, Aurora A, the kinase identified in this study as responsible for P-Ser^83^-HP1γ at G_2_/M, is amplified and overexpressed in cervical cancer, which induces centrosome amplification, aneuploidy and transformation [38]. Cervical cancer patients with high Aurora A expression correlate with a poorer disease-free survival and overall survival rates than patients with low Aurora A expression, indicating that this protein could be used as a prognostic marker [39]. Based on the current study, the high levels of both HP1γ and Aurora kinases in cervical cancer cells would suggest that there is a resultant increase in P-Ser^83^-HP1γ. Thus, targeting this pathway would affect P-Ser^83^-HP1γ-mediated cell proliferation, in addition to other downstream Aurora effectors. In fact, Aurora kinase inhibitors have been shown to suppress proliferation of cervical cancer cells and enhance chemosensitivity [40,41], suggesting that targeting Aurora in combination with the HP1-histone methyltransferase pathway may be a beneficial therapy in these patients.

## Conclusions

In summary, the current study identifies a novel Aurora-HP1γ pathway that involves P-Ser^83^-HP1γ by Aurora A in G_2_/M and localization of this HP1γ subpopulation to the spindle pole, which is necessary for proper cell division. Combined, these results constitute robust evidence that P-Ser^83^-HP1γ plays a role in mitosis and bears importance for understanding impairments, which have been shown to be characterized by abnormally high levels of HP1γ and Aurora kinase activity, including cancer. Our results also suggest a teleological interpretation, namely that certain regulators of chromatin dynamics and transcription, such as HP1γ, may undergo functional pressures (for example Aurora A phosphorylation) to maintain the integrity of cell division so that their own epigenetic inheritance is reproducible from cell generation to cell generation.

## Methods

### Cell lines, reagents and cell treatments

Cell lines were obtained from the American Type Culture Collection (ATCC, Rockville, MD, USA) and maintained according to the manufacturer’s protocol. The human LX2 cell line was obtained as a generous gift from Dr Steve Freeman (Mount Sinai, NY, USA). Roscovitine (Sigma-Aldrich, St Louis, MO, USA) treatment was added at increasing concentrations (0, 5, 10 and 20 μM) for 8 hours, and lysates were harvested. Cells were treated with 3 μg/ml aphidicolin or 2 μg/ml nocodazole (both from EMD Millipore, Billerica, MA, USA) for 16 hours to arrest at G_1_/S and G_2_/M, respectively. Control cells were treated with vehicle, dimethyl sulfoxide (DMSO). HeLa cells were synchronized by double thymidine block. Thymidine (2 mM, Sigma-Aldrich) was added to asynchronous cells for 18 hours. Cells were subsequently released for 9 hours in regular growth media prior to the second thymidine (2 mM) block. After 17 hours, cells were released for the thymidine block and lysates were collected at the indicated time points. KT5720 was obtained from EMD Millipore. MLN8237 and hesperidin were purchased from Selleckchem (Houston, TX, USA). For hesperidin treatment, HeLa cells were arrested in mitosis by treatment with nocodazole for 16 hours. Arrested cells were treated with 200 nM hesperidin for the indicated times in the presence of 10 μM of the proteasome inhibitor MG132 (Sigma-Aldrich) to prevent mitotic exit [28].

### Plasmids, siRNA and recombinant adenovirus

Standard molecular biology techniques were used to clone HP1γ into the pGEX and Ad5CMV vectors. For HP1γ-specific transient shRNA-mediated knockdown, complementary oligonucleotides were synthesized for the target sequence (GCAAATCAAAGAAGAAAAG), annealed and ligated into the pCMS3 vector (kindly provided by Dr Daniel Billadeau, Mayo Clinic, Rochester, MN, USA). For stable shRNA-mediated HP1γ knockdown, control or HP1γ-specific shRNA lentiviral particles (Santa Cruz Biotechnology, Inc, Santa Cruz, CA, USA) were used to infect cells according to the manufacturer’s protocol, followed by puromycin selection (2 μg/ml). Myc-tagged wild type and dominant negative constructs for Aurora A and Aurora B were a kind gift from Dr Paolp Sassone-Corsi [21]. S83A-HP1γ and S83D-HP1γ mutations were obtained using the QuickChange Site-Directed Mutagenesis Kit, as suggested by the manufacturer (Agilent Technologies, Inc, Santa Clara, CA, USA). All constructs were verified by sequencing at the Molecular Biology Core at Mayo Clinic, Rochester, MN, USA. Aurora A (*AURKA*) and Aurora B (*AURKB*) Silencer validated siRNAs were purchased from Ambion-Life Technologies (Carlsbad, CA, USA). Epitope-tagged (6xHis-Xpress) HP1γ, S83A-HP1γ and S83D-HP1γ, as well as EV (Ad5CMV), were generated as recombinant adenovirus in collaboration with the Gene Transfer Vector Core at the University of Iowa, IA, USA.

### Western blot analysis

Samples were run on 4 to 20% gradient SDS-PAGE gels (Lonza, Walkersville, MD, USA) or 12% SDS-PAGE gels and electroblotted onto polyvinylidene difluoride (PVDF) membranes (EMD Millipore). The membranes were blocked in 5% BSA in tris-buffered saline Tween-20 (TBST) for 1 hour at room temperature. The blots were incubated for 2 hours at room temperature or overnight at 4°C with primary antibody (P-Ser^83^-HP1γ [8], 1:1,000; HP1γ, 1:1,000; and P-Ser^10^-H3, 1:5,000 (all from EMD Millipore); Aurora A, 1:1,000 (BD Biosciences Pharmingen, San Diego, CA, USA); Aurora B, 1:500; cyclin B1, 1:1,000; cyclin B2, 1:1,000; and CDK1, 1:1,000 (Abcam, Cambridge, MA, USA); β-actin, 1:1,000; and α-tubulin, 1:1,000 (Sigma-Aldrich); c-Myc (9E10) for Myc-tagged proteins, 1:1,000 (Thermo Scientific, Rockford, IL, USA); and OMNI D8 for His-tagged proteins, 1:1,000 (Santa Cruz Biotechnology)). After repeated washes in TBST, horseradish peroxidase (HRP)-conjugated anti-rabbit or mouse IgG secondary antibody (1:5,000) was added for 1 hour at room temperature. Blots were developed by Pierce ECL chemiluminescent substrate (Thermo Scientific).

### Immunofluorescence and confocal microscopy

Immunofluorescence and confocal microscopy were performed as previously described [42]. The primary antibodies were used at the following dilutions: P-Ser^83^-HP1γ, 1:200; and γ-tubulin, 1:500 (Sigma-Aldrich); Aurora A, 1:50; and Aurora B, 1:50 (BD Biosciences Pharmingen); cyclin B1, 1:500; cyclin B2, 1:100; and CDK1, 1:40 (Abcam); and cyclin D3, 1:200 (Cell Signaling Technology, Danvers, MA, USA). For localization of P-Ser^83^-HP1γ during S-phase, EdU incorporation was combined with immunofluorescence. Prior to fixation, cells were incubated for 30 minutes in media containing 10 uM EdU. Subsequently, cells were processed for immunofluorescence, followed by EdU labeling using the Click-iT EdU Imaging Assay Kit (Invitrogen, Carlsbad, CA, USA) according to the manufacturer’s protocol. For mitotic aberrations, spindle poles were labeled by immunofluorescence with γ-tubulin and counterstained with 4',6-diamidino-2-phenylindole (DAPI) containing mounting media (Vector Laboratories, Burlingame, CA, USA). For each condition, at least 200 mitotic cells were analyzed to quantify mitotic aberrations.

### GST fusion protein purification and in vitro kinase assays

GST fusion protein purification was done as previously described [8]. For Aurora A and Aurora B in vitro kinase assays, HP1 fusion proteins (10 μg) were incubated with recombinant kinases (EMD Millipore) and 10 mM ATP (Sigma-Aldrich) for 10 minutes at 30°C, in either the supplied buffer (Aurora A) or buffer containing 50 mM Tris pH 7.5, 0.1 mM ethylene glycol tetraacetic acid (EGTA), and 15 mM dithiothreitol (DTT, Aurora B). Kinase reactions were terminated by the addition of SDS loading dye and then resolved by western blot as described above.

### Cell proliferation assay

Cell proliferation was measured by EdU incorporation using both fluorescence-activated cell sorting (FACS) and microscopy. Cells were infected with adenovirus carrying control, HP1γ, S83A-HP1γ or S83D-HP1γ vectors. Forty-eight hours post-plating, cells were pulsed with 10 μM EdU (Invitrogen) for 1 hour. Subsequently, cells were processed using the Click-iT EdU Flow Cytometry or Imaging Assay Kits (Invitrogen) according to the manufacturer’s protocols. EdU incorporation was measured by FACS analysis at the Mayo Flow Cytometry Research Core Facility, Rochester, MN, USA, or confocal microscopy. Each experiment was performed at least five different times in triplicate, expressed as means with standard error of mean (SEM) and statistical analyses were performed using unpaired *t*-test.

### Gene expression profiling, microarray analysis

Global gene expression profiling was carried out at the Microarrays Facility of the Research Center of Laval University, CRCHUL, QC, Canada, utilizing the Affymetrix Human Gene 1.0 ST arrays (28,869 well-annotated genes and 764,885 distinct probes). Intensity files were generated by Affymetrix GCS 3000 7G and the GeneChip Operating Software (Affymetrix, Santa Clara, CA, USA). Data analysis, background subtraction and intensity normalization was performed using robust multi-array analysis (RMA) [43]. Genes that were differentially expressed along with false discovery rate were estimated from *t*-test (>0.005) and corrected using Bayesian approach [44,45]. Data analysis, hierarchical clustering and ontology were performed with the oneChannelGUI to extend affylmGUI graphical interface capabilities [46], and Partek Genomics Suite, version 6.5 (Partek Inc, St Louis, MO, USA) with ANOVA analysis. Final fold changes were calculated as x = 2^log2value. Probes with *P* value <0.05 and fold change ± 2.2 among HP1γ versus EV, S83A-HP1γ versus EV, and S83D-HP1γ versus EV were selected for further analysis. For GO ANOVA, a minimum threshold of three genes and *P* <0.05 was used to identify significant functional groups. To validate the Affymetrix microarray, targets with significant alteration (*P* <0.05) were compared to the real-time data using an arbitrary cutoff of ± 2.2 fold change compared to EV control.

## Abbreviations

ANOVA: analysis of variance; aph: aphidicolin; ATCC: American Type Culture Collection; BSA: bovine serum albumin; CDK: cyclin-dependent kinase; CDK1: cyclin-dependent kinase 1; CHO: Chinese hamster ovary; con: control; CRCHUL: Centre de Recherche du Centre Hospitalier de l'Université Laval; DAPI: 4',6-diamidino-2-phenylindole; DMSO: dimethyl sulfoxide; DTT: dithiothreitol; EdU: 5-ethynyl-2´-deoxyuridine; EGTA: ethylene glycol tetraacetic acid; EV: empty vector; FACS: fluorescence-activated cell sorting; GO: Gene Ontology; GST: glutathione S-transferase; GUI: graphical user interface; H3K9me: histone H3 lysine 9 methylation; HcpA: *Dictyostelium discoideum*, AX4 chromo domain-containing protein; HP1: heterochromatin protein 1; HRP: horseradish peroxidase; noc: nocodazole; Orc2: origin recognition complex subunit 2; PKA: protein kinase A; P-Ser10-H3: phosphorylation of histone H3 at serine 10; P-Ser83-HP1γ: phosphorylation of HP1γ at serine 83; P-Thr288: phosphorylation of Aurora A at threonine 288; PVDF: polyvinylidene difluoride; RMA: robust multi-array analysis; RT-PCR: reverse transcriptase polymerase chain reaction; SEM: standard error of mean; Ser10: serine 10; Ser83: serine 83; shCTRL: shRNA control; shHP1γ: shRNA knockdown of HP1γ; shRNA: short hairpin RNA; siRNA: small interfering RNA; TBST: tris-buffered saline Tween-20.

## Competing interests

The authors declare that they have no competing interests.

## Authors’ contributions

RU and GL generated the main idea of the work and developed the study design, both conceptually and methodologically. AG, PL, SS, AJM, GU, EC and GL made substantial contributions to the acquisition of data. AG, PL, EC, JI, RU and GL contributed to analysis and interpretation of data. AG, RU and GL wrote the manuscript from first draft to completion. AG, PL, SS, AJM, GU, EC, JI, RU and GL made comments, suggested appropriate modifications and corrections. All authors read and approved the final manuscript.

## Supplementary Material

Additional file 1: Figure S1 (A) FACS-assisted cell cycle analysis of double thymidine block samples. HeLa cells were synchronized by double thymidine block and released for the indicated time points. Enrichment of cells is shown at the G_1_/S boundary 2 hours post-release, in S phase at 5 hours post-release and in mitosis at 8 hours post-release. (B) G_1_/S boundary peak of P-Ser^83^-HP1γ levels at 2 hours post-release from double thymidine block are diminished with PKA inhibition. PKA was inhibited with increasing concentrations of KT5720 as indicated upon release from double thymidine block and cell lysates were collected at 2 hours post-release. Pan-HP1γ levels are shown as a loading control. FACS, fluorescence-activated cell sorting; PKA, protein kinase A; P-Ser^83^-HP1γ, phosphorylation of HP1γ at serine 83. Click here for file

Additional file 2: Table S1 q-PCR array validation of Affymetrix Human Gene 1.0 ST microarray. Click here for file
