# Supplementary figures and images for: Functional impact of Aurora A-mediated phosphorylation of HP1γ at serine 83 during cell cycle progression
Source: Epigenetics Chromatin. 2013 Jul 5;6:21. doi: 10.1186/1756-8935-6-21 (PMC3707784; doi:10.1186/1756-8935-6-21)

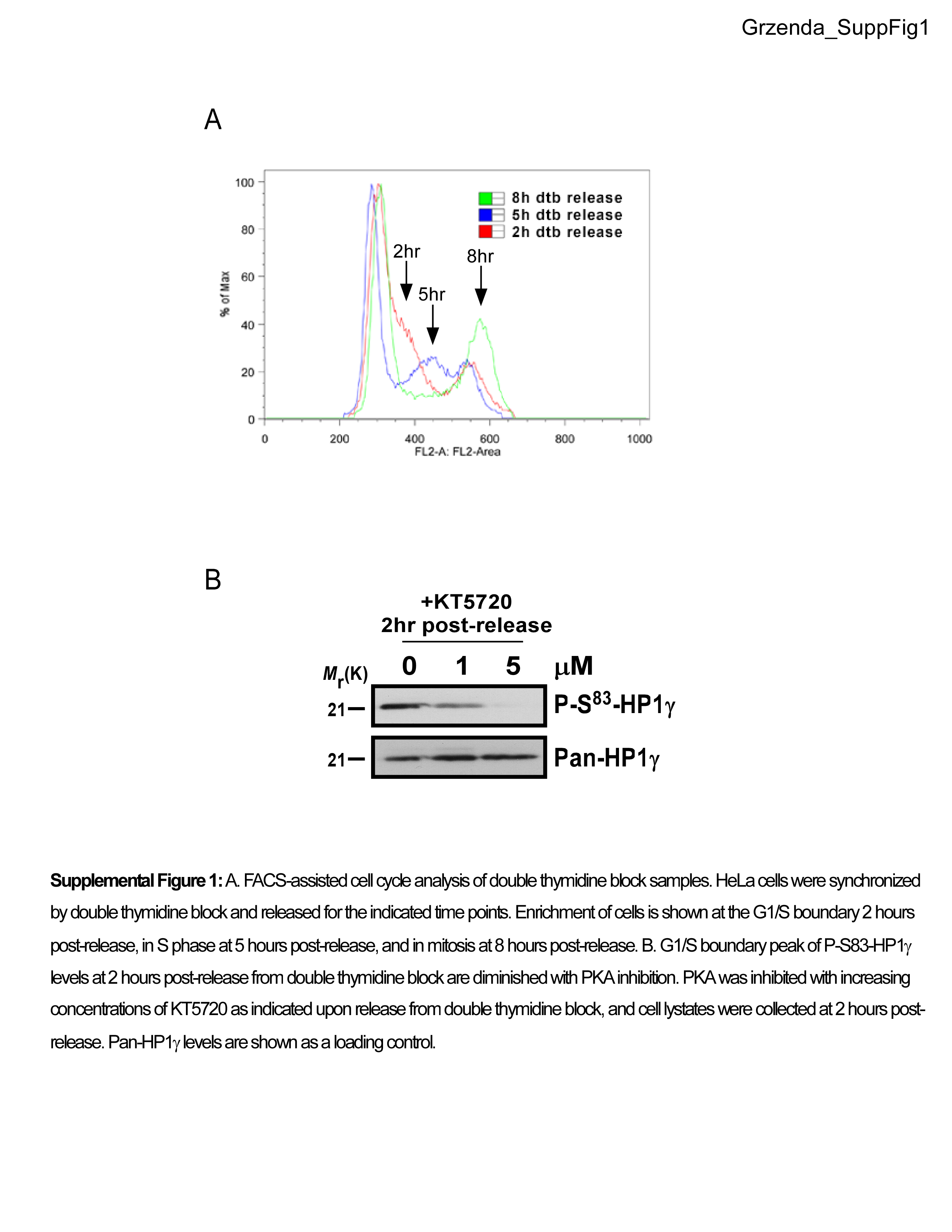

Supplement: Additional file 1: Figure S1 — (A) FACS-assisted cell cycle analysis of double thymidine block samples. HeLa cells were synchronized by double thymidine block and released for the indicated time points. Enrichment of cells is shown at the G1/S boundary 2 hours post-release, in S phase at 5 hours post-release and in mitosis at 8 hours post-release. (B) G1/S boundary peak of P-Ser83-HP1γ levels at 2 hours post-release from double thymidine block are diminished with PKA inhibition. PKA was inhibited with increasing concentrations of KT5720 as indicated upon release from double thymidine block and cell lysates were collected at 2 hours post-release. Pan-HP1γ levels are shown as a loading control. FACS, fluorescence-activated cell sorting; PKA, protein kinase A; P-Ser83-HP1γ, phosphorylation of HP1γ at serine 83. [file 1756-8935-6-21-S1.png]
