# Supplementary material for: Functional impact of Aurora A-mediated phosphorylation of HP1γ at serine 83 during cell cycle progression
Source: Epigenetics Chromatin. 2013 Jul 5;6:21. doi: 10.1186/1756-8935-6-21 (PMC3707784; doi:10.1186/1756-8935-6-21)
Supplement: Additional file 2: Table S1 — q-PCR array validation of Affymetrix Human Gene 1.0 ST microarray. [file 1756-8935-6-21-S2.doc]

**Supplemental Table 1: Q-PCR Array Validation of Affymetrix Human Gene 1.0 ST Microarray**

|  | **qPCR (vs EV) fold changes** | | | **Affymetrix (vs EV) fold changes** | | |
| --- | --- | --- | --- | --- | --- | --- |
| **Symbol** | **HP1γ** | **HP1γ-S83A** | **HP1γ-S83D** | **HP1γ** | **HP1γ-S83A** | **HP1γ-S83D** |
| ECM1 | 2.08 | 2.15 | 2.34 | 2.24 | 2.31 | 2.47 |
| EREG | 2.63 | 2.38 | 3.41 | 2.40 | 2.22 | 2.21 |
| IL1B | −2.45 | −2.46 | −3.42 | −2.36 | −2.19 | −2.81 |
| MMP3 | 3.37 | 2.00 | 2.69 | 2.64 | 2.34 | 2.27 |
| VEGFA | −2.39 | −2.29 | −2.29 | −2.22 | −2.30 | −2.50 |
